# Collective health behavior and face mask utilization during the COVID-19 pandemic in Oklahoma, USA

**DOI:** 10.1093/pubmed/fdac007

**Published:** 2022-04-04

**Authors:** Laura A Bray, Olivia Porter, Andrew Kim, Lori L Jervis

**Affiliations:** Center for Applied Social Research, University of Oklahoma, Norman, OK 73019, USA; Center for Applied Social Research, University of Oklahoma, Norman, OK 73019, USA; Center for Applied Social Research, University of Oklahoma, Norman, OK 73019, USA; Department of Anthropology, Center for Applied Social Research, University of Oklahoma, Norman, OK 73019, USA

**Keywords:** collective behavior, Covid-19, face mask use, mask ordinances, Oklahoma

## Abstract

**Background:**

Face mask use offers an important public health tool for reducing the spread of coronavirus disease 2019 (COVID-19), yet the politicization of COVID-19 has resulted in uneven adherence. This study assesses the effects of setting characteristics and the sociodemographic composition of crowds on group-level masking rates.

**Methods:**

We conducted 123 site observations of masking behavior at public locations across Oklahoma (USA) between June and September 2020. We used analyses of variance and t-tests to examine variation in masking and ordinary least squares regression to model the effect of setting and sociodemographic characteristics on site-level masking rates.

**Results:**

The masking rate across all sites averaged 34% but varied widely. Site-level masking rates were higher at metropolitan sites and sites with a store or municipal masking mandate. The masking rate at sites where women or older adults (60+) were the predominant group did not differ significantly from other sites. Ethnically diverse sites exhibited significantly higher masking rates compared with predominantly white sites. Findings indicate that setting characteristics explained a greater amount of variation in collective masking rates than sociodemographic differences.

**Conclusions:**

This study underscores the importance of place and policy for mask adherence. In the absence of state-level mandates, masking policies at a more local level may be effective.

## Introduction

Throughout most of the coronavirus disease 2019 (COVID-19) pandemic in the USA, public health officials recommended universal face masking to slow the spread of the severe acute respiratory syndrome coronavirus 2 virus.^1^ However, face mask use quickly became a marker of political identity,^2^ rather than a non-partisan public health tool.^3^^,^^4^ Many Republican governors refused to issue statewide mask mandates, contributing to high COVID-19 incidences in Republican-led states beginning in June 2020.^5^ In the absence of state directives, local businesses and municipalities implemented a patchwork of public health measures, including local and site mask mandates, within highly contentious environments.^6^

Face masking requires high levels of collective adherence to effectively limit the spread of COVID-19.^7^ Prior research identifies numerous predictors of individual-level masking, including geographic,^8^ sociodemographic^9^ and policy factors.^10^ However, fewer studies examine variation in masking at higher levels of aggregation, particularly the level of group interaction where disease transmission occurs. The objective of this study was to (1) examine variation in collective masking behavior across public sites in Oklahoma, and (2) assess the effect of site-level setting characteristics (urban–rural status, site type and masking policy) and sociodemographic characteristics on collective masking behavior.

## Methods

### Research setting

Oklahoma did not implement a statewide mask mandate during the pandemic but local municipalities enacted ordinances, despite facing controversy and challenges.^11^ Norman and Stillwater, the state’s two major college towns, adopted mask mandates in the second week of July 2020, followed by the cities of Tulsa and Oklahoma City about a week later.^12^^,^^13^ These ordinances generated intense controversy, including death threats and a recall attempt against the mayor in Norman^14^^,^^15^ and the retraction of Stillwater’s initial attempt at a mask ordinance in May 2020 after public outcry and threats of violence.^16^ A year into the pandemic, the few locales that had passed mask requirements (many of them urban centers) struggled with noncompliance from their mandate-less neighbors.^17^

In July 2020, a number of nationally based retailers enacted mandatory masking policies, including Sprouts Farmers Market, Walmart, Trader Joe’s, Whole Foods and Dollar General.^18–21^ Dollar Tree and Family Dollar initially required masks in July, then reversed the decision and ‘requested’ masks before re-implementing required masking in early August.^22^ Our observational data indicate that some local stores required masking earlier than their national chains. The Trader Joe’s in Oklahoma City, for example, implemented a mandate over 4 months before the national chain followed. The policies of some retailers (e.g. Dollar General) varied widely by site. As late as January 2021 some retail stores had not enacted mandatory masking policies (e.g. Hobby Lobby and Atwoods Farm Store). Oklahoma’s tribal casinos, by contrast, adopted mask mandates relatively early, generally on reopening in late May or June.^23–25^

### Study design

We conducted 123 site observations (2836 people) of public behavior with implications for the spread of COVID-19 between 13 June and 5 September 2020, as the pandemic escalated in Oklahoma.^26^ A team of four researchers used a checklist to document the presence or absence of a facial mask at observation sites by approximate age, gender and race. Most observed behavior consisted of persons entering and exiting indoor spaces, with occasional direct observations of the corresponding indoor spaces. We conducted repeat observations at a subsample of nine retail stores, before and after the implementation of a mask mandate.

### Measurements

#### Masking rate

Our outcome variable measured the proportion of masked persons out of total observed at each site.

#### Geography

We classified sites’ urban/rural status according to the US Department of Agriculture’s (USDA) rural–urban commuting area (RUCA) codes. RUCA codes use population density, urbanization and daily commuting to categorize geographic areas on a 1–10 scale.^27^ We collapse the scale into three categories: *metropolitan*, *micropolitan* and *small town/rural*.

#### Site type

Site types included (1) *retail* (e.g. grocery stores, dollar stores, gas stations), (2) *food and beverage services* (e.g. restaurants, bars, coffee shops), (3) *travel and leisure sites* (e.g. casinos, hotels, museums), (4) *outdoor recreation* (e.g. public parks, downtown areas) and (5) *other* (e.g. non-profit and religious sites).

#### Mask mandates

We gathered information about mask mandates at the municipal (town, city or county) and site (business or organization) levels through news outlets, government websites and company press releases, as well as signage at observation sites.

#### Demographics

Observers assigned persons into relatively broad gender and age categories (e.g. man, woman, other/undetermined; age 60 and older, under age 60). We characterized sites as composed of *predominantly women* or *predominantly men* when 60% or more of observed persons presented as the respective gender category. Similarly, we categorized crowds as *predominantly older* or *predominantly younger* based on whether 60% or more of observed persons appeared to be over or under age 60.

Because of the difficulty simultaneously documenting multiple demographic categories, observers noted the approximate racial/ethnic breakdowns of crowds rather than individual persons. Racial categories included White, American Indian, Black, Latinx and Asian American. We classified sites with ~60% or more White people as *predominately white* and all other sites as *ethnically diverse*.

### Statistical analysis

We used one-way analysis of variance (ANOVA) to determine if collective masking rates varied by geography, site type, gender or age. The ‘other’ category of site types was excluded from the ANOVA because of its small sample size (*n* = 2). T-tests for independent samples were used to examine masking patterns by mask mandates and race/ethnicity. A paired t-test compared masking rates before and after the implementation of mask mandates using the subsample of repeat observation. Based on the findings from the ANOVAs, we narrowed down relevant geographic, site type, gender and age variables for inclusion in the regression analysis. We then performed multivariate ordinary least squares (OLS) regression to model the relationship between collective masking rates and site characteristics.

Errors in data collection resulted in 12 missing values on the race variable. The one-way ANOVA by race/ethnicity excluded observations with missing data (*N* = 111). Prior to the regression analysis, we performed chained multiple imputation (*m* = 20) to fill the 12 missing values.^28^ The imputation process used all variables that appear in the subsequent regression analyses. The final regression models used Rubin’s formula to combine the estimates into a single set of regression parameters.^29^ For all statistics, we used a *P*-value of 0.05 to determine significance and report 95% confidence intervals (CI). We performed all analyses using Stata 17.

## Results

### Descriptive statistics

**Fig. 1:**
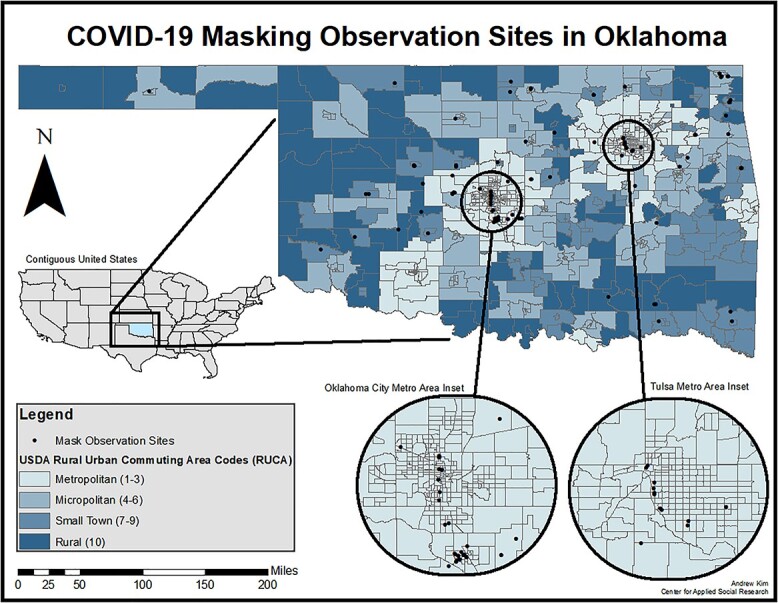
Map of COVID-19 masking observations sites by urban/rural status, Oklahoma (USA).

Research teams observed an average of 23 people per site over observations that averaged 7 min (Table 1). The bulk of observations occurred in northeastern and southcentral Oklahoma, areas with the largest population centers (Fig. 1). Metro locations comprised 54% (*n* = 66) of the sample, with micropolitan areas (*n* = 20, 16.3%) and small town or rural areas (*n* = 37, 30%) comprising the remainder. Most observations occurred at retail establishments (*n* = 88, 71.5%), followed by food and beverage services (*n* = 17, 13.8%), travel and leisure (*n* = 8, 6.5%), outdoor recreation (*n* = 8, 6.5%) and other sites (*n* = 2, 1.6%).

**Table 1:** Descriptive statistics of observation sites (*N* = 123)

	n/mean	Percent/SD	Range
Masking rate	34.0	35.3	0–100
Individuals observed/site	23.1	23.1	5–164
Observation length	7.1	5.7	0.5–40 min.
Geography			
Metro	66	53.7	0–1
Micropolitan	20	16.3	0–1
Small town and rural	37	30.1	0–1
Type of setting			
Retail	88	71.5	0–1
Food and beverage	17	13.8	0–1
Travel and leisure	8	6.5	0–1
Outdoor recreation	8	6.5	0–1
Other	2	1.6	0–1
Any mask mandate	29	23.6	0–1
Municipal mandate	26	21.1	0–1
Site mandate	25	20.3	0–1
Gender			
Predominantly women	28	22.8	0–1
Predominantly men	29	23.6	0–1
Mixed gender	66	53.7	0–1
Age			
Predominantly older (60 and older)	6	4.9	0–1
Predominantly younger (under 60)	106	86.2	0–1
Mixed age	11	8.9	0–1
Race and ethnicity^a^			
Predominantly white	86	69.9	0–1
Ethnically diverse	25	20.3	0–1

^a^ *N* = 111 due to missing data.

On the individual level, 43.9% of total people observed wore masks (*n*/*N* = 1,246/2,836). However, the collective masking rate for all 123 site observations averaged 34% (SD 35.3; CI 27.76–40.24) and varied considerably across locations, ranging from 0 to 100%. Observations largely occurred prior to the implementation of face mask mandates; only 29 sites (23.6%) had either a city or site policy at the time of the observation. Of these, 21 had both a city and site mandate, five had only a municipal mandate and four only a site mandate. Metro sites had higher rates of mask mandates: out of the metro sites, 27 (40.9%) had mask mandates compared with only two (3.5%) non-metro sites.

A total of 28 sites (22.8%) consisted of predominately women and 29 sites (23.6%) of men. Older adults predominated at six sites (4.9%) and younger people at 106 sites (86.2%). Most sites (*n* = 86, 69.9%) consisted of a predominantly white demographic. A total of 25 sites (20.3%) were ethnically diverse.

### Variation in collective masking behavior

Metro locations had the highest collective masking rates at 51.4%. Small town/rural and micropolitan locations had dramatically lower masking rates at 15.7 and 10.3%, respectively. Using a one-way ANOVA, we found statistically significant differences based on geography (*F*(2, 120) = 24.2; *P* < 0.001). A Tukey post hoc test revealed significantly higher masking rates at metro locations compared with micropolitan (41.16; CI 23.0–59.4; *P* > 0.001) and small town/rural locations (35.7; CI 21.1–50.4; *P* > 0.001). The difference between micropolitan and small town/rural sites did not reach significance.

Retail sites averaged the highest masking rate at 38.2%, followed by travel and leisure (34.0%), food and beverage (24.2%), and outdoor recreation sites (3.9%). ANOVA reveals a statistically significant difference in masking between site types (*F*(3, 117) = 3.0; *P* = 0.04). Masking rates were significantly lower at outdoor recreation sites compared with retail sites (34.3; CI 1.4–67.2; *P* = 0.04). The differences between other site types did not reach significance.

Sites with any mask mandate had dramatically higher masking rates (68.1%) compared with those without (23.0%). A t-test for independent samples showed that this difference is statistically significant (37.6%; CI 26.9–48.4%; *t*(121) = 7.2; *P* < 0.001). To further explore the relationship between policies and masking rates, we observed a subsample of nine retail stores in Norman and Oklahoma City before and after the enactment of mask mandates, including five grocery stores, a pharmacy, a liquor store and a farm/ranch store. Mask utilization increased from 58.5% in the pre-mask mandate observations to 86% following implementation a masking policy, a statistically significant change of 27.5% (CI 13.3–41.7; *t*(8) = 4.5, *P* = 0.002).

Sites comprised of predominantly men had lower average masking rates (24.9%) compared with majority women (35.9%) or mixed gender (37.2%) sites. However, ANOVA shows that these differences were not statistically significant (*F*(2,120) = 1.3; *P* = 0.28). Sites with large proportions of older adults exhibited higher rates of masking (50.9%) compared with those with younger people (33.9%) or mixed aged crowds (25.5%). The between-group differences based on age was not statistically significant (*F*(2, 120) = 1.0; *P* = 0.37). Finally, ethnically diverse sites had higher masking (49.0%) compared with predominantly white sites (32.1%), a statistically significant difference of 16.9% (CI 1.0–32.9; *t*(109) = 2.11, *P* = 0.04).

### Predictors of collective masking behavior

Our first regression model predicted site masking rates using setting characteristics (metro, outdoors and mask mandate; see Table 2). All three variables reached significance. Metropolitan settings were positively and significantly related to masking rates, with metro sites exhibiting 25.5% (CI 14.7–36.3) higher predicted masking compared with non-metro sites. Outdoor locations had a predicted 20.2% (CI 34.3 to 6.1) lower masking rate compared with indoor locations. The presence of a mandate also significantly predicted masking rates; rates at sites with a mandate were a predicted 30.2% (CI 15.3–45.1) higher compared with those without. Combined, setting characteristics explained ~42% of the variation in masking rates.

**Table 2:** OLS regression predicting masking rates by setting characteristics (*N* = 123)

	*β*	Robust SE	95% CI	*P*
Metro	25.48	5.45	14.69 to 36.27	0.00
Outside	−20.18	7.11	−34.26 to −6.10	0.01
Mask mandate	30.19	7.54	15.27 to 45.12	0.00
Constant	14.53	3.02	8.54 to 20.52	0.00
*R^2^*	0.42			

Our second regression model tested the effects of the sociodemographic composition of crowds (predominantly men, predominantly older and ethnically diverse) on collective masking rates (Table 3). None of the variables reached significance. The sociodemographic composition of sites explained about 7% of variation in masking rates.

**Table 3:** OLS regression predicting collective masking rates by sociodemographic composition of crowds (*N* = 123)

	*β*	Robust SE	95% CI	*P*
Predominantly men	−5.74	3.55	−12.78 to 1.29	0.11
Predominantly older	8.55	7.05	−5.41 to 22.51	0.22
Ethnically diverse	15.51	8.00	−0.33 to 31.35	0.06
Constant	32.47	4.05	24.44 to 41.00	0.00
*R^2^*	0.07			

**Note:** We filled 12 missing values on the diversity variable using multiple imputation. We used chained regression equations (*m* = 20) and included all variables that appear in the regression analyses. We combined the estimates using Rubin’s formula to obtain a single set of regression parameters.^29^

## Discussion

### Main findings of this study

Our study found an average collective masking rate of 34% across public sites in Oklahoma between 13 June and 5 September 2020. Although we found differences in site-level masking rates based on the sociodemographic composition of crowds, these differences generally failed to reach significance. Notably, multivariate regression showed that the race, gender and age variables combined explained only a small amount of the variation in masking rates at sites (7%). The variables measuring setting characteristics (metro, mask mandate and outdoors) by contrast explained 42% of the variation in collective masking. These findings were robust to different model and variable specifications (analyses not shown).

### What is already known on this topic

Most prior studies on face mask utilization focus on the individual level and establish the importance of geographic, sociodemographic and policy factors on masking decisions. Both survey and observational studies find relatively high masking rates among women, older adults and people of color,^30–38^ as well as urban residents.^8^^,^^9^ Research also shows that mask mandates effectively increase individuals’ likelihood of masking.^9^^,^^10^ However, we currently know less about how these factors translate into collective behavior.

A substantial amount of research examines demographic differences in individual masking behaviors and generally finds that gender, age and race significantly predict a person’s likelihood of masking.^9^^,^^30–33^^,^^35^^,^^38–44^ However, prior research at higher levels of aggregation—primarily the county and state levels—comes to more mixed conclusions. Several studies have used *The New York Times* survey data from July 2020 to explore predictors of county-level masking. Pro and colleagues found a significant relationship between collective masking and the percentage of residents who are female, older (65+) and non-white.^45^ Kahane^46^, by contrast, found no effect of gender and, out of several race and ethnicity measures, only the percentage of the population that is Hispanic reached significance. The author also found a relationship between older age groups and mask wearing. Cunningham and Night^47^, after controlling for contextual factors, found no association between masking rates at the county level and the percentage of female and older (age 65+) residents, but did find a significant effect of the percentage of non-white population.

### What this study adds

To our knowledge, this is the first masking study to employ direct observations to examine predictors of collective masking at the group level. Because social behavior is ‘collectively negotiated and context-dependent,’^48^ studies focused on the individual level alone may offer limited information about how people behave at specific times and places.^49^ Understanding how collective masking varies across sites of daily life is particularly important for places without statewide mandates, where more localized features of the environment may more strongly influence collective health behaviors. Our study adds to the growing body of literature that indicates distinct processes and predictors of COVID-19 masking behaviors at the individual and collective levels,^45–47^ and suggests the need for continued attention to how the micro-context shapes collective health behaviors.

### Limitations

Our study has several limitations. First, this study has limited ability to shed light on the impact of mask policies/mandates in rural communities, as only two of our non-metropolitan sites had such policies. However, the average masking rate at these non-metro sites (21.7%) was still higher than non-metro sites without a mandate (13.5%). Second, because our data collection was observational only, the underlying motivations and meaning of masking remain unknown. Third, our study did not account for variation in COVID-19 levels which may have influenced Oklahomans’ likelihood of masking.

## Conclusions

The surge of the COVID-19 Delta and Omicron variants in 2021 and renewed calls for universal masking speak to the continued relevance of masking as long as the virus is linked to significant public health impacts.^50^ Our findings suggest that policies at the local level can effectively increase masking rates in the absence of state directives. Our repeat observations show that local-level policies worked even in cities like Norman that experienced backlash following the implementation of masking mandates. Thus, this study points to the importance of face masking mandates at various levels during COVID-19 outbreaks and for the implementation of local level policies when higher level government leaders fail to put more far-reaching mandates into place.

## Data availability statement

The datasets generated and analyzed during the current study are available from the corresponding author on request.

## Funding

This work was supported by a University of Oklahoma Vice President for Research and Partnerships COVID-19 Rapid Response Seed Grant.

## Conflicts of interest

The authors have no relevant financial or non-financial interests to disclose.

## Notes

**Laura A. Bray**, Research Scientist

**Olivia Porter**, Research Assistant

**Andrew Kim**, Research Assistant

**Lori L. Jervis**, Professor
